# Medical Association of Delaware County, (Ohio)

**Published:** 1880-06

**Authors:** 


### MEDICAL ASSOCIATION OF DELAWARE COUNTY, (OHIO).

### Meeting of May 11, 1880.

The attendance was quite large, and the interest shown in the discussion marked the prosperity of the association.

Dr. W. McIntire, of Millville, presented a case of monstrosity. Mrs. E. W., æt. 25, married three years, had gone her full time. He was called and made an examination, but found no advancement in the case; gave an opiate and returned home; in about ten days was again called and then found an actually case of labor. He said he was unable to determine the presentation but found he had a partial shoulder presentation. He tried to bring the head into position, but failed to find it, yet thought he could feel the face. A few good pains soon brought away the child; on examination he found there was another child and which was soon born, but both dead. The last natural in every part, while the first he found fully developed except the vertical portion of the frontal bone, the parietal bones, the temporal, the occipital bone and the vertebral column down to the lumbar region. These portions were entirely wanting.

Prof. E. T. Nelson of the O. W. University, was present and delivered an interesting physiological lecture on monstrosities. Drs. E. H. Hyatt, McDowell and others spoke at some length endorsing the idea of pressure as the cause of arrested nutrition, rather than maternal imaginations on the fœtus in utero.

The subject of fractures of the upper extremities was taken up. Prof. E. H. Hyatt said of all the fractures of this region Colles fracture was the most difficult to treat satisfactorily. He had treated a good many cases both with the straight splint and the pistol-shaped splint. The most important point in the treatment he would call attention, to be used in applying the dressings, whether the dressing be plaster pa.is, pistol or straight splint, that was to prevent pressure over or on the annular ligament or on the wrist joint. He rather held to the pistol-shape splint for Colles fracture.

D. A. W. Dunn said his experience was small but he would endorse Dr. Hyatt.

Dr. McDowell favored the plaster dressing or starch bandage, as this could not be so easily removed, and was of the opinion better results could be obtained.

Dr. White had used the splint spoken of by Dr. H., but he generally made his own splints and used them straight. He generally left the hand more or less free, and by this limited motion prevented the stiffening of the joint so generally seen in those cases.

Dr. J. McCann reported a case of *trichiniasis* coming under his care three miles east of the city; Miss L. æt. 16. Last December Mr. S. killed some fattened hogs; salted the pork down, and some few days ago gave Mr. L. some of the pork for his family. The meat was roasted and the outside being well-cooked was given to the hired help for their dinner; and the young lady ate of that portion less cooked near the bone. At supper the meat was again cooked and all of the family ate of it. About four days after the girl was taken sick, with pains in the muscles, fever, delirium, etc., and diarrhœa. The family supposed the case one of malarial trouble and administered quinine; after some five days more Dr. McCann was sent for and at once suspected trichinia spiralis, and had some of the meat procured. The specimen was given Professor E. T. Nelson, who by means of the microscope showed the meat alive with these species of entozoa.

The association selected the following gentlemen delegates to the American Medical Association: Prof. E. H. Hyatt, Drs. Wm. McIntire, A. C. Dunn, and J. McCann; and the following gentlemen as delegates to the State Medical Society: Prof. E. H. Hya't, Drs. J. H. White, S. W. Fowler, E. A. Westbrook.

